# Physician Confidence in Artificial Intelligence: An Online Mobile Survey

**DOI:** 10.2196/12422

**Published:** 2019-03-25

**Authors:** Songhee Oh, Jae Heon Kim, Sung-Woo Choi, Hee Jeong Lee, Jungrak Hong, Soon Hyo Kwon

**Affiliations:** 1 Division of Nephrology Department of Internal Medicine Soonchunhyang University Hospital Seoul Republic of Korea; 2 Department of Urology Soonchunhyang University Hospital Seoul Republic of Korea; 3 Department of Orthopedic Surgery Soonchunhyang University Seoul Hospital Seoul Republic of Korea; 4 Department of Internal Medicine New York Medical College New York Health Hospital New York, NY United States

**Keywords:** artificial intelligence, AI, awareness, physicians

## Abstract

**Background:**

It is expected that artificial intelligence (AI) will be used extensively in the medical field in the future.

**Objective:**

The purpose of this study is to investigate the awareness of AI among Korean doctors and to assess physicians’ attitudes toward the medical application of AI.

**Methods:**

We conducted an online survey composed of 11 closed-ended questions using Google Forms. The survey consisted of questions regarding the recognition of and attitudes toward AI, the development direction of AI in medicine, and the possible risks of using AI in the medical field.

**Results:**

A total of 669 participants completed the survey. Only 40 (5.9%) answered that they had good familiarity with AI. However, most participants considered AI useful in the medical field (558/669, 83.4% agreement). The advantage of using AI was seen as the ability to analyze vast amounts of high-quality, clinically relevant data in real time. Respondents agreed that the area of medicine in which AI would be most useful is disease diagnosis (558/669, 83.4% agreement). One possible problem cited by the participants was that AI would not be able to assist in unexpected situations owing to inadequate information (196/669, 29.3%). Less than half of the participants(294/669, 43.9%) agreed that AI is diagnostically superior to human doctors. Only 237 (35.4%) answered that they agreed that AI could replace them in their jobs.

**Conclusions:**

This study suggests that Korean doctors and medical students have favorable attitudes toward AI in the medical field. The majority of physicians surveyed believed that AI will not replace their roles in the future.

## Introduction

Research into and usage of artificial intelligence (AI) has been gaining popularity in the field of computer science [1-3]. Recently, various kinds of AI programs have been developed based on “big data” collected through the Internet of Things. AI programs have been widely used in the manufacturing sector, the information-communications industry [4], and the medical field [5-7]. The development and utilization of AI programs in the medical field are currently entering the stage of commercialization [8,9]. AI is defined as the ability of computer systems to perform tasks that would usually require human levels of intelligence. A subfield of AI is machine learning, which can be used to teach a computer to analyze a vast amount of data in a rapid, accurate, and efficient manner through the use of complex computing and statistical algorithms [10,11].

In the past, it was thought that AI would replace doctors in many areas [12-15]. However, this has not occurred. Recent scientific advances have been rapid, suggesting that this might be a possibility. IBM’s Watson was developed from a huge database of published literature and millions of medical records [16,17]. Based on this, it can assist in the establishment of precise diagnoses and proper treatment plans [16,17]. Furthermore, Watson provides advice on the best treatments for cancer and conducts genome analyses [18]. Similarly, Google’s DeepMind software is being used to test the feasibility of the automated grading of digital fundus photographs using optical coherence tomography [19]. Recently, AI has been used to predict genetic variations in low-grade gliomas [20], identify genetic phenotypes in small cell lung carcinoma [21], decrease false-positive rates in screening mammography computer-aided detection [22], improve pathologic mediastinal lymph node detection [23], and automatically perform bone age assessment [24]. These examples demonstrate the influence of AI in medicine. The application of AI will be further extended to other areas in the future, leading to fundamental changes in the role of physicians and the way they practice medicine [25].

Korea is regarded as a technologically advanced country. Among people aged 18 to 24 years in the Republic of Korea, mobile phone penetration is 97.7%. Of the approximately 19 million households in Korea, 99.2% have internet access via an optical local area network, digital subscriber line (xDSL), cable modem, mobile device, or other media compared to approximately 75% in the United States [26].

There are differing perspectives on the future of AI. A pessimistic view of AI is that AI will replace humans in many industries. Optimistic views also exist in which humans will have more opportunities to benefit from clinical advances in the future with AI support [27]. Recently the AlphaGo AI program defeated a human Go professional, which shocked Korean society and provoked controversy in Korea [28,29].

Recent news reports revealed that Korean patients would follow AI advice over a doctor’s advice about their cancer treatment [30]. However, there is no research on the opinions and attitudes of Korean physicians toward the application of AI programs in the medical field. Current medical students and young physicians will be affected by AI before they retire. Therefore, physicians need to be prepared for these changes to use AI effectively as a tool.

The purpose of this study is to investigate the awareness of AI programs among Korean medical doctors and to provide basic information about physicians’ awareness of and reactions to the introduction of AI in the future.

## Methods

### Participants

This study was approved by an institutional review board at Soonchunhyang Medical College Hospital Seoul (no. 2017-05-014). Using Google Forms, we surveyed medical students, doctors who graduated from Soonchunhyang Medical College, and doctors at hospitals affiliated with Soonchunhyang University. The survey was administered online through a mobile phone invitation. Demographic and professional information on the medical students and doctors were obtained. Each participant was sent a unique link to the online survey. Participants were informed about the goal of the survey (medical research) in the preface of the questionnaire. By voluntarily participating in the survey after being given adequate information on its purpose, informed consent was implied. We confirm that participation was voluntary; participants could not be identified from the material presented and no plausible harm to participating individuals could arise from the study. Responses were made on a single Web page with one “submit” button that only allowed submissions through these unique links, thus making noninvited responses extremely unlikely.

### Measurement Instruments

#### Survey

In May 2017, our online survey, consisting of 11 closed-ended questions, was conducted (Textbox 1 and Multimedia Appendix 1). Survey content validity was reviewed by study researchers (n=5) and a panel of physicians (n=5) who were accepting patients at their sites. Following this, pilot testing was performed by medical college students (n=20) and physicians (n=80) who did not participate in developing the survey. Our survey was in accordance with the Checklist for Reporting Results of Internet E-survey (CHERRIES) [31]. The contents of the survey consisted of a questionnaire regarding the recognition of and attitudes toward AI, the direction of AI development in medicine, and the possible risks of using AI in the medical field. Three internal medicine physicians consulted the latest journals on AI and composed the questionnaire [5,6,9,16-18,32-34]. We sent 3000 doctors and medical students Web links to the questionnaire. These potential participants were almost entirely alumni of Soonchunhyang Medical College or were employed at hospitals affiliated with Soonchunhyang University.

The answers to five questions (Q1-Q5) were assessed using a five-point ordinal Likert scale (1=strongly disagree to 5=strongly agree). For three additional questions (Q6, Q8, Q10), 50 sample respondents were given to the questions in an open-ended format, and the five most commonly given answers were selected to be the five possible answer choices for survey participants.

**Questions asked in the online survey regarding artificial intelligence (AI) in the medical field. The answers to questions 1-5 were assessed with a five-point Likert scale (1=strongly disagree to 5=strongly agree).**

**Attitudes**

Q1. Do you agree that you have good familiarity with artificial intelligence?

Q2. Do you agree that artificial intelligence has useful applications in the medical field?

Q3. Do you agree that the diagnostic ability of AI is superior to the clinical experience of a human doctor?

Q4. Do you agree that artificial intelligence could replace your job?

Q5. Do you agree that you would always use AI when making medical decisions in the future?

Q6. What are the advantages of using artificial intelligence?

- AI can speed up processes in health care
- AI can help reduce medical errors.
- AI can deliver vast amounts of clinically relevant high-quality data in real time
- AI has no space-time constraint
- AI has no emotional exhaustion nor physical limitation

Q7. If your medical judgment and an artificial intelligence’s judgments differ, which will you follow?

- Doctor’s opinion
- Artificial intelligence’s opinion
- Patients’ choice

**Expected Applications in Medicine**

Q8. In which field of medicine do you think artificial intelligence will be most useful?

- Making a diagnosis
- Making treatment decisions
- Direct treatment (including surgery)
- Biopharmaceutical research and development
- Providing medical assistance in underserved areas
- Development of social insurance program

Q9. Which sector of health care do you think will be the first to commercialize artificial intelligence?

- Public primary care such as public health centers
- Primary care in private clinics
- Specialized clinics (spine, knee, obstetrics and gynecology, etc)
- University hospitals

**Possible Risks**

Q10. What are you concerned about application of AI in medicine? It cannot be used to provide opinions in unpredicted situations due to inadequate

- Information
- It is not flexible enough to be applied to every patient
- It is difficult to apply to controversial subjects
- The low ability to sympathize and consider the emotional well-being of the patient
- It is developed by a specialist with little clinical experience in medical practice

Q11. Who do you think will be liable for legal problems caused by artificial intelligence?

- Doctor in charge
- Company that created the artificial intelligence
- Patient who consented to follow artificial intelligence’s input

#### Questionnaire

1. Attitudes: the first part of the survey asked about the physician’s attitude toward the medical application of AI. The questions and possible answer choices (if applicable) are detailed in Multimedia Appendix 1. A total of seven closed-ended questions were included (Q1-Q7).
2. Expected applications in medicine: medical students and physicians were asked about the medical fields in which AI could be applied. The questions and possible answer choices are detailed in Multimedia Appendix 1. A total of two closed-ended questions were included (Q8 and Q9).
3. Possible risks: medical students and physicians were asked which problems they were concerned about regarding the application of AI in medicine. It is not clear who is liable when there are adverse clinical outcomes between humans and AI; therefore, we included a question about liability for AI decisions in medicine. The questions and possible answer choices for each question are detailed in Multimedia Appendix 1. A total of two closed-ended questions were included (Q10 and Q11).

#### Subgroup Analyses: Specialty, Working Status, and Medical Experience

We investigated whether attitudes differed regarding the medical applications of AI depending on the respondent’s specialty degree of medical experience, working status, and work location.

For this study, the categories for department were medical student, physician, surgeon, or other. The categories for working status were medical student, training physician (intern, resident, or clinical fellow), university professor, or nonuniversity physician. The categories for amount of medical experience were the number of years licensed from medical school graduation: less than 10 years, between 10 and 20 years, or more than 20 years. The categories for working location were in and around Seoul, large cities outside of Seoul, small cities outside of Seoul, or small towns or rural areas.

### Statistical Analysis

Basic statistics (mean and standard deviation or total number and percent) were computed for all covariates. In the subgroup analysis, Kruskal-Wallis tests served for evaluating the effect of gender factors of questionnaire items. The differences in the questionnaire responses according to working state, location, licensed years, and medical specialty were analyzed using the Mann-Whitney test. For all tests, the level of significance was set at *P* ≤.05.

## Results

### Participants

During the study period, 669 participants, out of approximately 3000, completed the survey (22.3% rate of return). There were 121 medical students, 162 training physicians, and 386 physicians. Among these participants, 22.4% (150/669) were younger than 30 years, and 22.1% (148/669) were female. The demographic and professional characteristics of the participants are listed in Table 1.

### Questionnaire

The results of the questionnaire are summarized in Table 2.

### Responses to the Questionnaire

#### Attitudes

**Figure 1:**
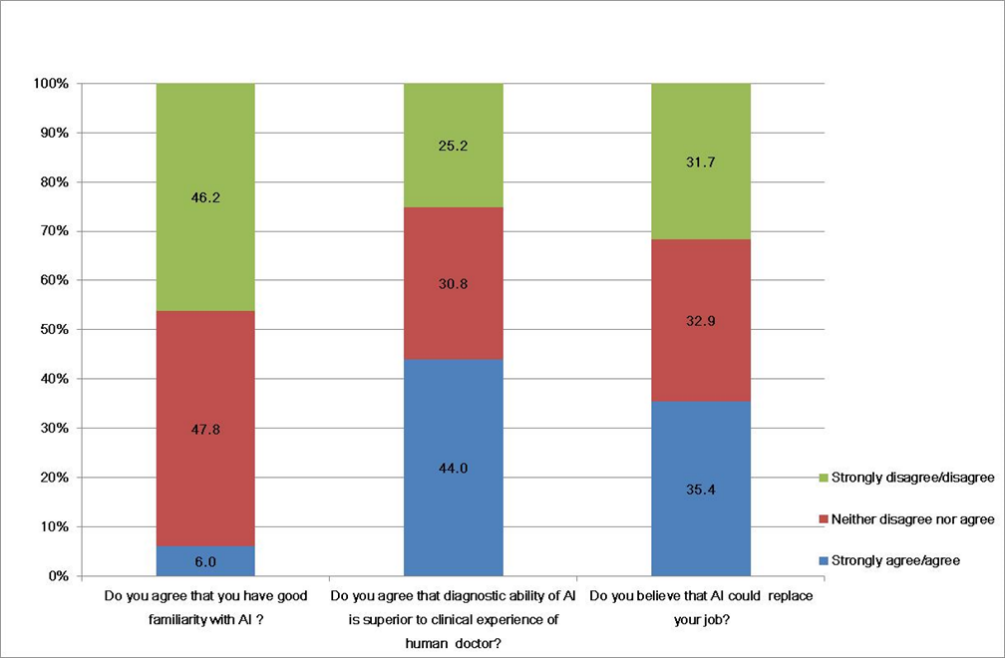
Major results of the questionnaire. AI: artificial intelligence.

**Figure 2:**
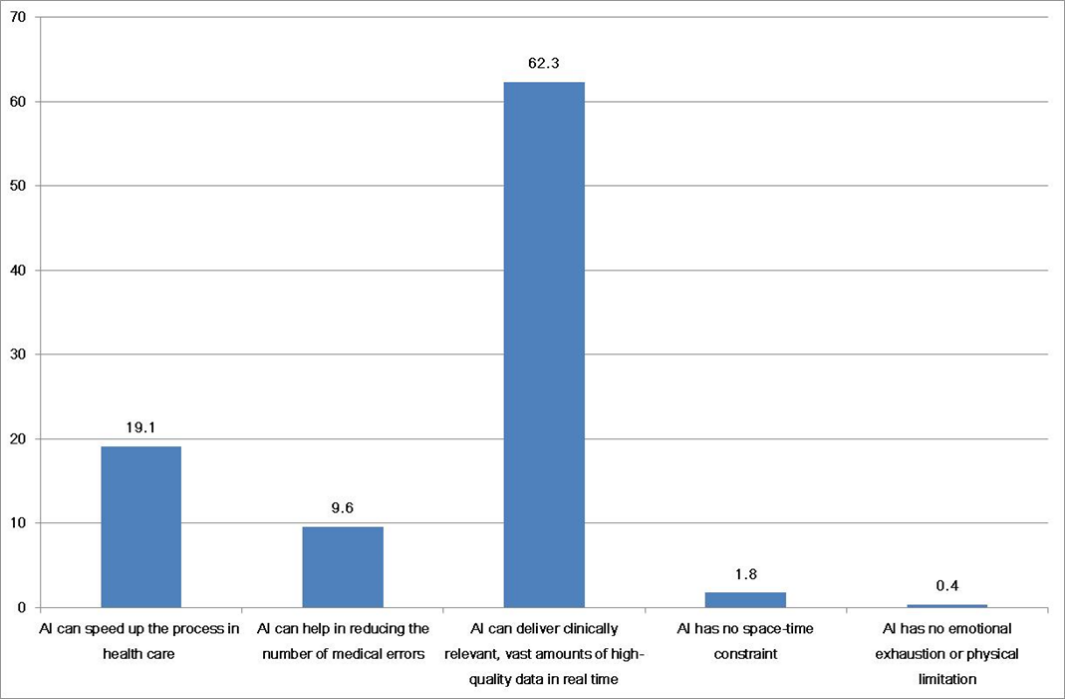
Responses about the advantage of artificial intelligence (AI) in medicine.

Generally, familiarity with AI was low. Only 40 of 669 respondents (6.0%) answered that they had good familiarity with AI (Figure 1). Many participants considered AI useful in the medical field (73.4%, 558/669). The respondents agreed that the advantages of using AI were its ability to quickly obtain vast amounts of clinically relevant, high-quality data in real time (62.3%, 417/669), speed up processes in health care (19.1%, 128/669), and decrease the number of medical errors (9.6%, 64/669) (Figure 1). However, fewer than half of the participants agreed that “AI is superior to a doctor’s experience” (44%, 294/669), “AI could replace a doctor” (35.4%, 237/669), or “AI would be used whenever medical decisions need to be made” (42%, 281/669) (Figures 1 and 2). If there were differences between an AI’s decision and a doctor’s opinion regarding a medical decision, 79% (528/669) of participants would follow the doctor’s opinion. The results from the attitudes section of the questionnaire are summarized in Table 2.

#### Expected Application in Medicine

Respondents felt the areas in medicine where AI would be most useful in the future were reaching a diagnosis (83.4%, 558/669) and forming a treatment plan (53.8%, 360/669). Fewer than 10% felt it would be useful in providing medical assistance in underserved areas (9.6%, 64/669), treating patients independently (eg, performing surgery, 9.0%, 60/669), or developing medical insurance guidelines (6.1%, 41/669). Additionally, most participants (66.2%, 443/669) thought that AI would be first commercialized at a university hospital. The results from the expected fields section of the questionnaire are summarized in Table 2.

#### Possible Risks

According to the respondents, the possible problems with AI are that AI would be unable to provide an opinion in an unpredicted situation owing to inadequate information (29.3%, 196/669) and that it would not be applied to every patient (34.1%, 228/669). In the case of a medical problem caused by AI, respondents felt responsibility should lie with the doctors (49.3%, 330/669), patients who consented to the use of AI (31.2%, 209/669), and the company that created the AI (19.4%, 130/669). The results from the possible risks section of the questionnaire are summarized in Table 2.

### Subgroup Analysis

The results of subgroup analysis according to the demographic characteristics of participants are summarized in Table 3.

**Table 1:** Demographic characteristics of participants surveyed about physicians and artificial intelligence (N=669).

Characteristics		n (%)
**Age (years)**		
	<30	150 (22.4)
	31-40	197 (29.4)
	41-50	159 (23.8)
	51-60	137 (20.5)
	61-70	18 (2.7)
	≥71	8 (1.2)
**Gender**		
	Male	514 (76.8)
	Female	148 (22.1)
	No response	7 (1.0)
**Working status**		
	Medical student	121 (18.1)
	Training physicians (intern, residents, fellows)	112 (16.7)
	University professors	90 (13.5)
	Nonuniversity physicians	346 (51.7)
**Licensed years**		
	Medical student	121 (18.1)
	<10 years	177 (26.5)
	10-20 years	170 (25.4)
	>40 years	201 (30.0)
**Medical specialty**		
	Medical student	121 (18.1)
	Medical department	284 (42.5)
	Surgical department	204 (30.5)
	Extra department	60 (9.0)
**Hospital status**		
	Medical school	121 (18.1)
	University hospital	162 (24.2)
	District general hospital	67 (10.0)
	Solo practice	217 (32.4)
	Group practice	30 (4.5)
	Long-term care hospital	24 (3.6)
	Community health center or military hospital	29 (4.3)
	Others	19 (2.8)
**Location of the clinics**		
	Seoul (Capital city)	278 (41.6)
	Seoul Metropolitan Area (Capital area)	162 (24.2)
	Regional Metropolitan City	44 (6.6)
	Cities	128 (19.1)
	Rural	57 (8.5)

**Table 2:** Participant’s attitudes on artificial intelligence (AI), the expected applications in medicine, and possible risks (N=669).

Question				n (%)	
**Attitudes**				
	**Q1. Do you agree that you have good familiarity with artificial intelligence?**			
		Strongly agree/agree		40 (6.0)
		Neither disagree nor agree		320 (47.8)
		Strongly disagree/disagree		309 (46.2)
	**Q2.** **Do you agree that AI has useful applications in the medical field?**			
		Strongly agree/agree		558 (73.4)
		Neither disagree nor agree		97 (14.5)
		Strongly disagree/disagree		14 (2.1)
	**Q3. Do you agree that the diagnostic ability of AI is superior to the clinical experience of human doctors?**			
		Strongly agree/agree		294 (44.0)
		Neither disagree nor agree		206 (30.8)
		Strongly disagree/disagree		169 (25.2)
	**Q4. Do you agree that AI could replace you in your job?**			
		Strongly agree/agree		237 (35.4)
		Neither disagree nor agree		220 (32.9)
		Strongly disagree/disagree		212 (31.7)
	**Q5. Do you agree that you will always use AI to make medical judgments in the future?**			
		Strongly agree/agree (=always/often)		281 (42.0)
		Neither disagree nor agree (=occasionally)		87 (13.0)
		Strongly disagree/disagree (=never/seldom)		301 (45.0)
	**Q6. What are the advantages of using AI?**			
		AI can speed up the process in health care		128 (19.1)
		AI can help in reducing the number of medical errors		64 (9.6)
		AI can deliver clinically relevant, vast amounts of high-quality data in real time		417 (62.3)
		AI has no space-time constraint		12 (1.8)
		AI has no emotional exhaustion or physical limitation		3 (0.4)
	**Q7. If your judgment and AI judgments differ, which will you follow?**			
		Doctor’s opinion		528 (78.9)
		Artificial intelligence’s opinion		110 (16.4)
		Patients’ choice		31 (4.6)
**Expected fields**				
	**Q8. In which field of medicine do you think AI will be most useful?**			
		Making diagnoses		558 (83.4)
		Making the decision for treatment		360 (53.8)
		Direct treatment (including surgery)		60 (9.0)
		Biopharmaceutical research and development		84 (12.6)
		Provide medical assistance in underserved areas		64 (9.6)
		Development of social insurance program		41 (6.1)
	**Q9. Which sector of health care do you think will be the first to commercialize AI?**			
		Public primary care such as public health centers		98 (14.6)
		Primary care in private clinics		31 (4.6)
		Specialized clinics (spine, knee, obstetrics, and gynecology, etc)		97 (14.5)
		University hospitals		443 (66.2)
**Possible risks**			
	**Q10.** **Which problems are you concerned about regarding the application of AI in medicine?**			
		It cannot be used to provide opinions in unexpected situations owing to inadequate stored information		196 (29.3)
		It is not flexible enough to be applied to every patient		228 (34.1)
		It is difficult to apply to controversial subjects		38 (5.7)
		Low ability to sympathize and consider the emotional well-being of the patient		179 (26.8)
		It was developed by a specialist with little clinical experience in medical practice		19 (2.8)
	**Q11. Who do you think will be responsible for medical problems caused by AI?**			
		Doctor in charge		330 (49.3)
		Company that created the artificial intelligence		130 (19.4)
		Patients who agreed to follow artificial intelligence’s input		209 (31.2)

#### Specialty

There was no significant difference in attitude toward AI according to the department of the respondent for any of the questions (Q1-Q5).

#### Working Status

There were no significant differences according to working status for three questions (Q2, Q4, Q5). There were significant differences for two questions (Q1, Q3). For the question about the recognition of AI (Q1), the outcomes for training physicians are summarized in Table 4.

#### Amount of Medical Experience

There was no statistical difference according to the degree of medical experience for three questions (Q2, Q4, Q5). There were significant differences between two questions (Q1, Q3). For the question about the recognition of AI (Q1), the outcomes for physicians licensed for less than than 10 years, physicians licensed between 10 and 20 years, and physicians licensed more than 20 years were higher than for medical students. For the question about the superiority of AI in diagnostic ability (Q3), the outcomes of medical students and physicians licensed for fewer than 10 years were higher than for physicians licensed between 10 and 20 years and physicians licensed more than 20 years. The results of the subgroup analysis according to the amount of medical experience and age are summarized in Table 4. Age showed a similar result as the analysis according to license year.

#### Working Location

There was no significant difference in attitudes toward AI according to working location.

**Table 3:** Subgroup analysis according to the demographic characteristics of participants.

Question	*P* value^a^				
	Department	Working status	License year	Age	Location
Q1. Familiarity of AI^b^	.06	<.001	<.001	<.001	.54
Q2. Usefulness of AI	.07	.11	.24	.24	.10
Q3. Diagnostic ability of AI	.38	.001	<.001	<.001	.07
Q4. Replacement human job (doctor)	.46	.19	.35	.32	.52
Q5. Frequency of using AI	.92	.95	.92	.43	.17

^a^ *P* values for categorical variables are based on Kruskal-Wallis tests.

^b^AI: artificial intelligence.

**Table 4:** Subgroup analysis according to working status.

Subgroup and question			Median (IQR^a^)	*P* value^b^	Post hoc
**Working status**					
	**Q1. Familiarity with AI^c^**			<.001	A<B, C, D
		A. Students	2 (2-3)		
		B. Training physician	3 (2-3)		
		C. Professor	3 (2-3)		
		D. Clinical physicians	3 (2-3)		
	**Q3. Diagnostic ability of AI**			.001	A=B>C=D
		A. Students	4 (3-4)		
		B. Training physician	4 (3-4)		
		C. Professor	3 (2-3)		
		D. Clinical physicians	3 (2-4)		
**License year**					
	**Q1. Familiarity with AI**			<.001	A<B=C=D
		A. Students	2 (2-3)		
		B. <10 years	3 (2-3)		
		C. 10-20 years	3 (2-3)		
		D. >20 years	3 (2-3)		
	**Q3. Diagnostic ability of AI**			<.001	A=B>C=D
		A. Students	4 (3-4)		
		B. <10 years	4 (3-4)		
		C. 10-20 years	3 (2-4)		
		D. >20 years	3 (2-4)		
**Age**					
	**Q1. Familiarity with AI**			<.001	A<B=C=D=E
		A. 20-29 years	2 (2-3)		
		B. 30-39 years	3 (2-3)		
		C. 40-49 years	3 (2-3)		
		D. 50-59 years	3 (2-3)		
		E. >60 years	3 (2-3)		
	**Q3. Diagnostic ability of AI**			<.001	A=B>C=D=E
		A. 20-29 years	3 (3-4)		
		B. 30-39 years	4 (3-4)		
		C. 40-49 years	3 (2-4)		
		D. 50-59 years	3 (2-4)		
		E. >60 years	3 (2-4)		

^a^IQR: interquartile range.

^b^*P* values for categorical variables are based on Mann-Whitney tests.

^c^AI: artificial intelligence.

## Discussion

### Principal Results and Comparison With Prior Work

To the best of our knowledge, this study is the first survey of the attitudes of physicians toward AI. The results of this survey suggest that the recognition of AI by medical students and doctors is low. However, they regarded AI to be useful in the medical field. Physicians and medical students felt that AI would be most useful for reaching a diagnosis and formulating a treatment in the future. The majority of Korean doctors do not believe that AI will replace them.

Precision medicine is “an emerging approach for disease treatment and prevention that takes into account individual variability in genes, environment, and lifestyle for each person” [35]. This approach allows doctors to choose treatment and prevention strategies for their patients. It requires significant computing power and algorithms that can learn by themselves at an unprecedented rate. Therefore, there is no precision medicine without AI. In our study, most physicians expected that AI would be helpful with diagnoses and in planning treatment by providing the latest clinically relevant data.

We asked the participants about the diagnostic superiority of AI compared to that of doctors. Fewer than half of the participants agreed that AI would be diagnostically superior. In the subgroup analysis, doctors in academic positions and office clinicians who had more clinical experience were less likely than medical students and training physicians to agree that AI is diagnostically superior. Additionally, experienced clinicians (licensed for more than 10 years) were less likely to agree that AI has superior diagnostic ability. Our questions were about general clinical practice. Although pathologists and radiologists were among the respondents, there were relatively few (27/669, 4.0%).

In contrast to our study, recent studies have shown that image recognition technology might make predictions or recognize diseases as effectively as or even better than physicians [17,36]. Liu and colleagues [36] from Google used an AI technique called convolutional neural network machine learning and demonstrated that AI achieves image-level area under the curve scores greater than 97% on both the Camelyon16 test set (metastasis detection of lymph nodes) and an independent set of 110 slides compared to a human pathologist, who achieved 73.2% sensitivity. Metastasis detection is currently performed by pathologists when reviewing large expanses of biological tissue. This process is labor intensive and error prone. However, AI machine learning saves time and is less likely to make errors [37]. In the case of radiology and pathology, some believe that AI will replace doctors based on diagnostic superiority [9,25,38]. Furthermore, AI could be able to extract fine information about tissues invisible to the human eye and process these data quickly and accurately [39,40].

Generally, AI has been used in imaging and pathology and is considered favorably in these fields [41-45]. Pathology and radiology have a common destiny as “informational specialists” with regard to images and pathology [38]. However, we did not investigate the reasons for their choices. They might believe that technical progress in the field of AI will not reach the level of human intelligence. It is also possible that Korean physicians have not examined the recent data on AI in the medical field.

In our study, 35.4% of participants agreed that doctors will be replaced by AI. This is not consistent with previous studies about AI. A 2017 survey by the Pew Research Center conducted with 4135 participants found that the public is roughly twice as likely to express worry (72%) than enthusiasm (33%) about a future in which robots and computers are capable of doing many human jobs [46]. Unlike other occupations, doctors felt that there would be difficulties in replacing doctors. Krittanawong [40] argued that AI cannot replace doctors yet at the bedside, given its limitations. First, AI cannot engage in high-level conversation with patients to gain their trust, reassure them, or express empathy [47]. These are all important parts of the doctor-patient relationship. Second, although AI sensors may glean valuable information to help with diagnosis, physicians will still be needed for interpretation in ambiguous situations to integrate medical histories, conduct physical exams, and facilitate further discussion [40]. It is possible that many Korean doctors believe this intuitively.

Skepticism can arise when applying AI to medical care. Regulations and principles of AI application need to be defined. AI can provoke ethical and legal problems in medicine. A regulatory authority should control AI algorithms for public safety. This issue will require debate from a social perspective.

Our survey response rate was 22.3%, which seems to be a lower response rate. However, previous studies demonstrated that electronic modalities often have lower response rates than paper mailed surveys [48-50]. Internet-based surveys demonstrated a lower response rate (45%) than the mail questionnaires (58%) (absolute difference 13%, 95% confidence interval 4%-22%, *P*<.01) [48]. A Cochrane review of randomized controlled trials identified numerous methods to increase response rates for both postal and electronic surveys [51]. With reference to this study, we made the questionnaire short, used a simple header, and gave a deadline. However, there were no monetary incentives, one of the major factors that increase the response rate. Plus, we could not provide a prenotification nor send reminder messages or have follow-up contact because we could not distinguish between those who already responded and the nonresponders.

### Limitations

Some limitations of our study should be noted. First, we did not ask background questions concerning how much the individual participants technically understood AI. Each participant may have had different conceptualizations of AI. Second, there is the possibility of selection bias. Participants may have been more motivated and might have expressed more positive attitudes compared to nonparticipants. Because the data were self-reported, a bias owing to social desirability cannot be excluded. In addition, the selected participants may not have been a good representation of Korean doctors in general. However, our study did include various ages and clinical backgrounds. Third, the questionnaire about AI was created by doctors rather than AI experts.

### Conclusion

This study found that physicians felt the application of AI to medicine would be useful. Physicians felt that the areas in medicine where AI would be most useful were diagnosis and treatment planning. However, more than half of the physicians did not believe AI would replace their role as health care providers. From a diagnostic point of view, doctors who had more experience favored a physician’s experience over AI. Follow-up surveys and multinational studies should be conducted to further explore these issues.

## Abbreviations

AI: artificial intelligence
IQR: interquartile range

## Appendix

Multimedia Appendix 1 Online survey form.
