# Supplementary material for: Physician Confidence in Artificial Intelligence: An Online Mobile Survey
Source: J Med Internet Res. 2019 Mar 25;21(3):e12422. doi: 10.2196/12422 (PMC6452288; doi:10.2196/12422)
Supplement: Multimedia Appendix 1 [file jmir_v21i3e12422_app1.pdf]

Korean version of Google survey

https://docs.google.com/forms/d/e/1FAIpQLSdWUjMv9Kfnp38JKFkkN2XOSAHIy8ZcqRxCffCvnbuP3UUgXg/viewform

English version of Google survey

https://docs.google.com/forms/d/e/1FAIpQLScEVUaMyWz5DWUNvOmTQndNW-MsQi-M1hqRhZQo2WMwizyQA/viewform

Supplement Table 1. Questionnaire for attitudes toward medical application of artificial intelligence

**Question**				
**Q1. Do you agree that you have good familiarity with artificial intelligence?**				
☐ Strongly agree	☐ agree	☐Neither disagree nor agree	☐ disagree	☐Strongly disagree
**Q2. Do you agree that artificial intelligence has useful applications in the medical field?**				
☐ Strongly agree	☐ agree	☐Neither disagree nor agree	☐ disagree	☐Strongly disagree
**Q3. Do you agree that the diagnostic ability of AI is superior to the clinical experience of a human doctor?**				
☐ Strongly agree	☐ agree	☐Neither disagree nor agree	☐ disagree	☐Strongly disagree
**Q4. Do you agree that artificial intelligence could replace your job?**				
☐ Strongly	☐ agree	☐Neither disagree nor	☐	☐Strongly

agree		agree	disagree	disagree
**Q5. Do you agree that you would always use AI when making medical decisions in the future?**				
☐ Strongly agree	☐ agree	☐Neither disagree nor agree	☐ disagree	☐Strongly disagree
**Q6. What are the advantages of using artificial intelligence?**				
☐ AI can speed up processes in health care<br>☐ AI can help reduce medical errors.<br>☐ AI can deliver vast amounts of clinically relevant high-quality data in real time<br>☐ AI has no space-time constraint<br>☐AI has no emotional exhaustion nor physical limitation				
**Q7. If your medical judgment and an artificial intelligence's judgments differ, which will you follow?**				
☐ Doctor's opinion<br>☐ Artificial intelligence's opinion<br>☐ Patients' choice				
**Q8. In which field of medicine do you think artificial intelligence will be most useful?**				
☐ Making a diagnosis<br>☐ Making treatment decisions<br>☐ Direct treatment (including surgery)<br>☐ Biopharmaceutical research and development<br>☐ Providing medical assistance in underserved areas				

☐ Development of social insurance program
**Q9. Which sector of healthcare do you think will be the first to commercialize artificial intelligence?**
☐ Public primary care such as public health centers<br>☐ Primary care in private clinics<br>☐ Specialized clinics (spine, knee, obstetrics and gynecology, etc.)<br>☐ University hospitals
**Q10. What are you concerned about application of AI in medicine?**
☐It cannot be used to provide opinions in unpredicted situations due to inadequate information<br>☐ It is not flexible enough to be applied to every patient<br>☐ It is difficult to apply to controversial subjects<br>☐ The low ability to sympathize and consider the emotional well-being of the patient<br>☐ It is developed by a specialist with little clinical experience in medical practice
**Q11. Who do you think will be liable for legal problems caused by artificial intelligence?**
☐ Doctor in charge<br>☐ Company that created the Artificial Intelligence<br>☐ Patient who consented to follow Artificial Intelligence's input
